# Social capital and health among older adults in South Africa

**DOI:** 10.1186/1471-2318-13-100

**Published:** 2013-09-28

**Authors:** Shandir Ramlagan, Karl Peltzer, Nancy Phaswana-Mafuya

**Affiliations:** 1 HIV/AIDS/STIs and TB (HAST), Human Sciences Research Council, Pretoria, Port Elizabeth, South Africa; 2 Department of Psychology, University of Limpopo, Turfloop, South Africa; 3 ASEAN Institute for Health Development, Mahidol University, Salaya, Thailand; 4 Office of the Deputy Vice Chancellor, Nelson Mandela Metropolitan University, Port Elizabeth, South Africa

**Keywords:** Social capital, Self-reported health, Depressive symptoms, Cognitive functioning, Physical inactivity, Older adults, South Africa, WHO SAGE

## Abstract

**Background:**

Little is known about social capital and health among older adults in South Africa. This study investigates the association between social capital and several health variables, namely: self-rated health, depressive symptoms, cognitive functioning and physical inactivity, among older South Africans.

**Methods:**

We conducted a national population-based cross-sectional study with a national probability sample of 3840 individuals aged 50 years or older who participated in the Study of Global Ageing and Adults Health (SAGE wave 1) in 2008 in South Africa. Measures included socio-demographic characteristics, health variables, cognitive functioning and physical activity. Social capital was assessed with six components, namely: marital status, social action, sociability, trust and solidarity, safety, and civic engagement.

**Results:**

The social capital assessment revealed that 56% of the respondents were married or cohabiting, 45% reported low (0) social action, 42% reported medium (2–3) sociability, 43% reported high (2) trust and solidarity, 50% reported high (2–4) civic engagement and 42% reported medium (6) psychological resources. In multivariate analysis, self-reported good health was associated with younger age, having secondary education and higher social capital (being married or cohabiting, high trust and solidarity and greater psychological resources). Depressive symptoms were associated with lower social capital (not being married or cohabiting, lack of high trust and solidarity and low psychological resources). Better cognitive functioning was associated with younger age, higher educational level, greater wealth and higher social capital (being married or cohabiting, high trust and solidarity, lack of safety, higher civic engagement and greater psychological resources). Physical inactivity was associated with older age and lower social capital (lower social action, lack of safety, lower civic engagement and poorer psychological resources).

**Conclusions:**

Given the basis of these findings on cross sectional data and subsequent limitation, it was found that these study findings mimic the findings of many European and American studies. Social capital among the elderly generation in South Africa is imperative for better health.

## Background

Social scientists, policy makers and international institutions such as the World Health Organization and the World Bank purport that social capital contributes to health inequalities within and between populations [1]. There are many definitions of social capital [2-4] but most overlap. Social capital is a way of describing social relationships within societies or groups of people [5]. The most accessible definition and conceptualisation of social capital used in the health sciences originates with its founder, Putnam [5,6]. Putman [5] wrote that social capital consisted of five principal characteristics, namely: (1) community networks, voluntary, state, personal networks, and density; (2) civic engagement, participation, and use of civic networks; (3) local civic identity—sense of belonging, solidarity, and equality with other members; (4) reciprocity and norms of cooperation, a sense of obligation to help others, and confidence in return for assistance; (5) trust in the community. To put it simple, social capital is seen as the nature and the extent of networks one engages in and its associated norms of exchange [6,7]. As such, “social capital enables individuals to gain access to resources such as ideas, information, money, services, as well as favours and to have accurate expectations regarding the behaviour of others by virtue of their participation in relationships that are themselves the product of networks of association. This occurs as individuals elect to engage in various activities with others in order to pursue their leisure, familial, ethnic, local environments, or wider political interests” [7], p. 654. Within social capital, the concepts of bonding and bridging social capital were recently introduced [7]. Bonding social capital as the name suggests, refers to a close trusting relationship between a group/network of individuals who share a similar identity while bridging social capital is the relationship between people and groups who know that are not alike [7]. Given the above, Szreter and Woolcock [7] wrote that health outcomes “can be improved by expanding the quality and quantity of bonding social capital (among friends, family and neighbours) and bridging social capital (trusting relations between those from different demographic and spatial groups)” (p.655) [7].

There has been substantial international empirical evidence about associations between social capital and self-rated health [7-10]. However the literature is still strongly disputed in terms of the nature and scope of these associations [11,12]. A previous examination of self-rated health in Ireland revealed marked social gradients with health being related to age, marital status, tenure, educational status, social class, household size and eligibility for General Medical Services [13]. The health of individuals and in most studies, self-rated health, has been linked both positively and negatively to social capital [14-18]. Social disconnectedness [19] and perceived isolation [19,20] were associated with lower levels of self-rated health, while participating in activities was seen to increase health by three per cent [15]. Depression in one’s later life has been identified as one of the most common mental health problems affecting older people [21]. In a comparative study between five Mediterranean and seven non-Mediterranean countries, it was seen that people in Mediterranean countries lived close to their larger families, had more children within the household and there was more assistance exchange yet these people were more lonely and women more depressed [20]. Among older Americans it was seen that stress of daily life, the onset of depression as well as their negative effects can be mitigated by one’s social network but conversely, one’s social ties can themselves be a source of stress and thus contribute to a poor mental state [15,22].

It was also found that European female elders exhibit more depressive symptoms and that symptoms of depression also emerged as negatively correlated with social involvement leading one to believe that if older adults increased their social networks, depressive feelings would be reduced [21]. Depression later in life was reported to frequently coexist with medical illnesses and disability [21]. The lack of social interaction was also shown to negatively impact other people’s cognitive functioning [23,24]. Poor social connections, infrequent participation in social activities and social disengagement predict the risk of cognitive decline in older individuals [23]. Further, interaction with larger social networks was a marker that leads to less cognitive decline [24].

However, current evidence is inadequate to inform the development of specific social capital interventions to combat mental illness [23]. More studies are needed to strengthen the evidence base for this. Putnam in his work in America entitled it “Bowling Alone”. He shared his concerns that less American individuals join associations and participate together in a range of activities [6,7]. Bowling is generally regarded as a social activity yet this social lifestyle (bowling alone) was attributed to having “been socialized into suburban sprawl (driveways from the road into garages and no walkways between homes) and long commutes (less time in the neighbourhood), the advent of dual careers (and over-working at that), and over-reliance on the television as a (vastly inferior) substitute for local social interaction” (p.654) [7]. These and other studies show that meaningful bonding and bridging social interaction was necessary for the elderly to retain their cognitive functioning [7]. South Africa’s history of apartheid and labour migration has influenced contemporary life [25]. The Apartheid Group Areas Act and the labour migration system divided African families by recruiting young men and women to centers of employment, including mining, farming, and urban areas [25,26]. Restrictions on the movement and settlement of those not employed meant that most children, unemployed younger adults, and older people were required to live in rural or periurban areas [25,26]. Older people facilitated the economic migration of younger adults by caring for their grandchildren and safeguarding the family property [8]. In South Africa, rapid social change and the transition to democratic government have been transforming family relations, increasing intergenerational tensions and adaptations of the patriarchal family structures and community life [25,27].

Research on individual social capital and physical activity has tended to focus on the association between physical activity, generalized trust, and social participation [28]. Less is known about the association between network social capital, i.e., the resources accessed through one’s social connections, and physical inactivity [28]. Limited studies that have been conducted have found a link between social networks and physical inactivity [16,28]. This makes sense as the older one gets, the less activities they are able to fully participate compared to when they were younger. It was also found that higher network diversity was associated with a decreased likelihood of physical inactivity [28]. They also found that individuals who did not participate in any formal associations were more likely to be physically inactive compared to those with high levels of participation [28]. Network diversity mediated the association between physical inactivity and participation [28]. Generalized trust and the network components of reach and range were not shown associated with physical inactivity [28].

Overall, little is known about associations between social capital and various health variables among older adults in South Africa. Thus, this study investigates the association between social capital and several health variables, namely: self-rated health, depressive symptoms, cognitive functioning and physical inactivity, among older South Africans who participated in the Study of Global Ageing and Adults Health (SAGE wave 1) in 2008. This study will not only strengthen existing inconclusive international empirical evidence but will also provide much needed baseline information on the association between social networks and health status in South Africa, upon which policy and programming can be developed.

## Methods

### Sample and sampling procedure

We conducted a national population-based cross-sectional study with a sample of 3840 aged 50 years or older in South Africa in 2008. The SAGE sample design entailed a two-stage probability sample that included national estimates stratified by locality type (urban and rural), and by population group (including Black, Coloured, Indian or Asian and White). “The first stage of sampling was the selection of primary sampling units (PSUs), using the 2002 Human Sciences Research Council (HSRC) master sample as the sampling frame. The master sample is a probabilistic sample of 1 000 enumeration areas (EAs) drawn from the South African National Census, conducted by Statistics South Africa (SSA) in 2001. An EA is the smallest geographical unit allocated to a single enumerator during census enumeration. It constitutes a small piece of land for an enumerator to administer a questionnaire during a national population census. The size of most EAs is between 100 and 250 visiting points (VPs). A VP is a separate (non-vacant) residential stand, address, structure or flat in a block of flats or homestead. It is a dwelling and therefore often, but not always, corresponds to a household. For the SAGE study, 600 EAs were drawn from the master sample and used as PSUs. This stage of selection was done centrally at the HSRC. The master sample was stratified by province, residence and race, and the EAs were then selected with a probability proportional to size, with the estimated number of people aged ≥50 years in each EA as a measure of size. Therefore, EAs with a larger number of people aged ≥50 years had a higher chance of being selected.” [29], p.29.

“The second stage of the sample design was the selection of VPs – in this case, households – which formed the secondary sampling units. This stage involved plotting the locations of households on geo-referenced aerial photograph maps of urbanised areas. From these photographs, the co-ordinates of each household in the selected EAs were extracted (using ArcView 3.3 geographical information system software) and were used to create a list of households. The household list was updated as necessary after a field visit. Once households had been systematically selected from the updated listing, Garmin eTrex global positioning system (GPS) receivers were used to navigate to the households.” [29], p.29. The individual response rate among those aged 50 years or older was 77%. SAGE was carried out in South Africa in partnership between the WHO, the National Department of Health (NDOH), and the HSRC. The study was approved by the HSRC Ethics Committee and the NDOH. Written informed consent for participation in the study was obtained.

### Measures

*Social capital* was assessed with six components [30], see Additional file 1: 1. being married or cohabiting (vs. Never married, divorced, separated, widowed); 2. *Social action* was assessed with 4 items, e.g., “How often in the last 12 months have you worked with other people in your neighbourhood to fix or improve something?” Response options ranged from 1 = never to 5 = daily. Responses were dichotomised into 1 = at least once or twice per months or more and 0 = less than once a month. Cronbach alpha of this 4 item social action index was 0.77 in this sample.

3. *Sociability* was assessed with 4 items, e.g., “How often in the last 12 months have you been in the home of someone who lives in a different neighbourhood than you do or had them in your home?” Response options ranged from 1 = never to 5 = daily. Responses were dichotomised into 1 = at least once or twice per months or more and 0 = less than once a month. Cronbach alpha of this 4 item sociability index was 0.66 in this sample.

4. *Trust and solidarity* was assessed with 5 items, e.g., “Generally speaking, would you say that most people can be trusted or that you can’t be too careful in dealing with people?” Response options differed from “yes” and “no” to 1 = to a very great extent to 5 = to a very small extent. All responses were dichotomised into 1 = yes or to a great or very great extent and 0 = no or neither great nor small extent to a very small extent. Cronbach alpha of this 4 item trust and solidarity index was 0.63 in this sample.

5) *Safety* was assessed with 3 items, e.g., “In general, how safe from crime and violence do you feel when you are alone at home?” Response options ranged from 1 = completely safe to 5 not safe at all and “yes” and “no”. All responses were dichotomised into 1 = yes or completely or very safe and 0 = no or moderately to not safe at all. Cronbach alpha of this 3 item safety index was 0.64 in this sample.

6) *Civic engagement* was assessed with 3 items, e.g., “How much say do you have in getting the government to address issues that interest you?” Response options included here 1 = unlimited say to 5 = no say at all and “yes” and “no”. All responses were dichotomised into 1 = yes or unlimited or a lot to say and 0 = no or some to no say at all. Cronbach alpha of this 3 item civic engagement index was 0.64 in this sample.

*Psychological resources* were assessed with two items from the Perceived Stress Scale [31], e.g., “In the last month, how often have you felt that you were unable to control the important things in your life?” Response options ranged from 0 = never to 5 = very often. Items were reverse scored, in order to get a sense of control [31]. Cronbach alpha of this 2 item psychological resources index was 0.91 in this sample.

*Subjective health status* was assessed with one question: “In general, how would you rate your health today?” Response options ranged from 1 = very good to 5 = very bad. This was dichotomised into 1 = very good or good and 0 = moderate to very bad.

*Depressive symptoms* were assessed by one question, “Overall in the last 30 days, how much of a problem did you have with feeling sad, low or depressed?” Response option ranged from 1 = none to 5 = extreme. Those who responded with severe or extreme were classified as having depressive symptoms.

*Cognitive capacity*. A battery of cognitive tests was used to measure cognitive performance, in order to measure objective indicators of various aspects of cognition. The cognitive tests included verbal recall, verbal fluency and digit span (forward and backward). The three cognitive tests, which together measure concentration, attention and immediate memory. Respondents were asked to produce as many words (names of animals) as possible in a one-minute time span. This test measures the ability to retrieve information from semantic memory [32]. In the Verbal Fluency Test, each interviewee was asked to produce as many words as possible in a given category (animals), within a fixed time. The variables of interest are the number of correctly named animals. Immediate and delayed verbal recall: The person administering the test presented 10 words verbally, repeating the words three times to saturate the learning curve. After about 10 minutes, the respondent was asked to recall as many of the 10 words as possible, to test delayed recall and recognition. Thus, verbal recall scores indicate the average number of words recalled out of the 10 words presented. This test assesses learning capacity, memory storage and memory retrieval [33]. Digit span (forward and backward): For the forward test, participants are read a series of digits (for example, “8, 3, 4”) and must immediately repeat them back. If they recall the numbers correctly, they are given a longer series of digits, until failure. In the backward test, the person must repeat the numbers read to them, but in reverse order. The length of the longest list a person can remember in this fashion was that person’s digit span and was an estimate of working memory [34,35]. Overall cognition: The tests selected – word list recall, verbal fluency and digit span – accurately measure the cognitive domains most affected by impairment and the early stages of dementia. The overall cognitive score consists of a summation of the results, and converted to a scale of 0 to 100, where 0 represents worst cognition and 100 best cognitive functioning. The overall cognitive score was dichotomised by using the median into 48 or more (=1) and less than 48 (=0).

*Physical activity* was measured using the General Physical Activity Questionnaire (GPAQ). The instrument gathered information on physical activity in three domains (activity at work, travel to and from places, and recreational activities), as well as time spent on sitting. The questionnaire also assessed vigorous and moderate activities performed at work and recreational activities. Information on the number of days in a week spent on different activities and time spent in a typical day for each activity was also recorded [36]. For physical activity, in addition to the total minutes of activity, the activity volume was also computed by weighing each type of activity by its energy requirement in metabolic equivalents (METs). The number of days and total physical activity MET minutes per week were used to classify respondents into three categories of low, moderate, and high levels of physical activity. Physical inactivity was defined as those who had low levels of physical activity; moderate and high levels of physical activity were collapsed in further analysis [37].

*Economic or wealth status:* Wealth levels were generated through a multi-step process, where the asset ownership was converted to an asset ladder, Bayesian post-estimation method was used to generate raw continuous income estimates, and then transformed into Quintiles [38]. Income data are not easy to obtain accurately in household surveys; however, the indicators of income need to be estimated because of the important interrelationship between health and wealth. Therefore, permanent income estimates derived from household assets and characteristics of the dwelling retirement and retirement benefits, financial security, income, consumption and financial transfers were used [38].

### Data analysis

The data were entered using CSPro and analysed using STATA Version 10. The data was weighted using post-stratified individual probability weights based on the selection probability at each stage of selection. Individual weights were post-stratified by province, sex and age-groups according to the 2009 Medium Mid Year population estimates from Statistics South Africa [39]. Associations between the key outcomes of self-rated health, depressive symptoms, cognitive capacity, physical inactivity and socio-demographic variables were evaluated calculating odds ratios (OR). Multivariable logistic regression was used for the evaluation of the impact of explanatory variables for the outcomes self-rated health, depressive symptoms, cognitive capacity, physical inactivity (dependent variables). All variables statistically significant at the P < .05 levels in bivariate analyses were included in the multivariable models. In the analysis, weighted percentages are reported. Both the reported 95% confidence intervals and the P value are adjusted for the multi-stage stratified cluster sample design of the study.

## Results

### Descriptive results

The total sample included 3840 of 50 years or older South Africans, 44.1% men and 55.9% women; almost half (49.9%) were between 50 to 59 years old (see Table 1). The educational level of most participants (71.6%) was lower than secondary school education and an almost equal number of respondents (41%) reported low and high wealth. Within the age categories, we see that as age increases, self-rated good health, cognitive functioning, physical activity dropped. Interestingly, depressive symptoms remain about the same as age increases from 60 to 79 years old and are highest for those aged between 50 to 59 years old at 52%. More females than males reported depressive symptoms and physical inactivity and more of those with higher educational level reported good health and cognitive functioning. Physical inactivity remained the same through all educational levels except for primary education which was slightly higher. Wealthier people reported good health, cognitive functioning, physical inactivity yet less reported depressive symptoms.

**Table 1:** Sample characteristics and prevalence of health variables among older South Africans

**Variables** ***Sociodemographics***	**Total sample** **N (%)**	**Self-rated good health** **%**	**Depressive symptoms** **%**	**Cognitive functioning** **%**	**Physical inactivity** **%**
*All*	3840	37.9	49.9	52.0	60.5
*Age*					
50–59	1695 (49.9)	45.0	51.7	61.0	53.2
60–69	1233 (30.6)	33.6	48.0	47.5	65.4
70–79	661 (14.0)	27.6	47.8	40.1	70.3
80 and over	251 (5.5)	24.2	49.3	23.1	73.7
*Gender*					
Female	2202 (55.9)	34.6	52.1	47.4	63.1
Male	1638 (44.1)	42.1	47.1	57.8	57.2
*Educational level*					
No schooling	854 (25.2)	37.0	49.9	42.8	59.7
Less than primary	803 (24.0)	30.0	53.9	42.7	59.5
Primary	779 (22.4)	31.7	53.8	49.7	64.4
Secondary	923 (28.3)	51.2	43.3	75.2	59.6
*Wealth*					
Low	1482 (40.6)	31.3	51.6	41.3	59.8
Medium	731 (18.2)	37.2	52.7	45.1	58.1
High	1608 (41.2)	44.7	47.1	65.0	62.3
***Social capital variables***					
Married/cohabitating	2007 (55.9)	43.4	45.8	60.2	58.2
Never married/divorced/widowed	1762 (44.1)	30.9	55.3	41.8	63.5
*Social action*					
Low (0)	1816 (45.1)	35.6	52.1	47.2	66.8
Medium (1)	769 (24.3)	42.8	51.9	55.1	54.1
High (2–4)	1018 (30.6)	37.2	46.7	56.4	51.6
*Sociability*					
Low (0–1)	744 (19.2)	28.2	52.8	45.7	64.7
Medium (2–3)	1513 (42.2)	38.4	46.8	52.9	57.7
High (4)	1320 (38.6)	42.1	52.4	54.0	58.0
*Trust and solidarity*					
Low (0)	462 (16.0)	27.2	63.5	36.0	68.8
Medium (1)	1453 (40.7)	32.8	52.7	49.1	64.5
High (2)	1224 (43.3)	47.3	42.5	59.3	54.5
*Safety*					
Low (0)	323 (9.6)	33.3	62.1	64.2	49.2
Medium (1)	2364 (61.7)	33.8	49.6	48.4	64.2
High (2–3)	908 (28.7)	49.3	47.4	55.3	50.9
*Civic engagement*					
Low (0)	366 (9.0)	33.2	44.5	35.5	76.3
Medium (1)	1504 (41.5)	37.7	50.9	51.0	58.0
High (2–4)	1626 (49.5)	40.2	49.5	56.2	55.1
*Psychological resources*					
Low (2–5)	895 (25.9)	19.4	72.2	36.6	70.8
Medium (6)	1655 (42.0)	39.9	45.8	53.3	60.2
High (7–10)	1075 (32.1)	50.2	38.6	62.2	48.1

In terms of social capital, 56% were married or cohabiting, 45% reported low (0) social action, 42% reported medium (2–3) sociability, 43% reported high (2) trust and solidarity, 50% reported high (2–4) civic engagement and 42% reported medium (6) psychological resources (see Table 1). Table 1 also shows that more married/cohabitating respondents reported good health and cognitive functioning.

### Predictors of health outcomes

In bivariate analysis younger age, male gender and having secondary education and greater social capital (being married or cohabiting, medium sociability, high trust and solidarity, high safety and greater psychological resources) were associated with self-reported good health. In multivariate analysis younger age and having secondary education and greater social capital (being married or cohabiting, high trust and solidarity, and greater psychological resources) were associated with self-reported good health. Further, in bivariate analyses being female and lower social capital (not being married or cohabiting, lack of trust and solidarity, lack of safety and low psychological resources) were associated with depression symptoms. In multivariate analysis, lower social capital (not being married or cohabiting, lack of trust and solidarity and low psychological resources) was associated with depression symptoms (see Table 2).

**Table 2:** Odds ratios for self-reported good health and depressive symptoms among older South Africans

**Variables**	**Self-rated good health**		**Depression symptoms**	
***Sociodemographics***	**Unadjusted OR (95% CI)**	**Adjusted OR (95% CI)**	**Unadjusted OR (95% CI)**	**Adjusted OR (95% CI)**
*Age*				
50–59	1.00	1.00	1.00	---
60–69	0.62 (0.48-0.80)***	0.65 (0.48-0.87)**	0.86 (0.65-1.15)	
70–79	0.46 (0.28-0.76)**	0.49 (0.29-0.82)**	0.86 (0.64-1.14)	
80+	0.39 (0.19-0.80)*	0.55 (0.24-1.29)	0.91 (0.56-1.48)	
*Gender*				
Female	1.00	1.00	1.00	1.00
Male	1.37 (1.04-1.81)*	1.01 (0.74-1.38)	0.82 (0.67-1.00)*	1.06 (0.82-1.38)
*Educational level*				
No schooling	1.00	1.00	1.00	---
Less than primary	0.73 (0.51-1.05)	0.82 (0.54-1.25)	1.18 (0.88-1.57)	
Primary	0.79 (0.49-1.28)	0.92 (0.58-1.45)	1.17 (0.82-1.66)	
Secondary	1.79 (1.13-2.84)*	1.63 (1.06-2.07)*	0.77 (0.55-1.08)	
*Wealth*				
Low	1.00	---	1.00	---
Medium	1.30 (0.76-2.23)		1.05 (0.57-1.93)	
High	1.78 (0.97-3.25)		0.84 (0.42-1.68)	
***Social capital***				
Married/cohabiting	1.71 (1.37-2.13)***	1.48 (1.06-2.07)*	0.68 (0.53-0.88)**	0.68 (0.51-0.91)*
*Social action*				
Low	1.00	---	1.00	---
Medium	1.36 (0.86-2.14)		0.99 (0.70-1.41)	
High	1.07 (0.79-1.47)		0.91 (0.46-1.40)	
*Sociability*				
Low	1.00	1.00	1.00	---
Medium	1.58 (1.00-2.49)*	1.44 (0.88-2.35)	0.79 (0.56-1.11)	
High	1.85 (0.85-4.02)	1.54 (0.82-2.87)	0.99 (0.60-1.62)	
*Trust and solidarity*				
Low	1.00	1.00	1.00	1.00
Medium	1.31 (0.73-2.34)	1.13 (0.72-1.77)	0.64 (0.37-1.11)	0.74 (0.47-1.18)
High	2.40 (1.19-4.86)*	1.89 (1.09-3.30)*	0.42 (0.21-0.85)*	0.49 (0.27-0.88)*
*Safety*				
Low	1.00	1.00	1.00	1.00
Medium	1.02 (0.62-1.69)	1.19 (0.69-2.05)	0.60 (0.37-0.98)*	0.57 (0.31-1.03)
High	1.94 (1.18-3.18)**	1.65 (0.96-2.84)	0.55 (0.29-1.05)	0.80 (0.44-1.43)
*Civic engagement*				
Low	1.00	---	1.00	---
Medium	1.22 (0.65-2.31)		1.29 (0.78 -2.12)	
High	1.36 (0.75-2.47)		1.22 (0.75-1.97)	
*Psychological resources*				
Low	1.00	1.00	1.00	1.00
Medium	2.76 (1.79-4.26)***	2.22 (1.45-3.41)***	0.32 (0.19-0.55)***	0.34 (0.21-0.56)***
High	4.20 (2.33-7.59)***	3.18 (1.69-6.00)***	0.24 (0.12-0.47)***	0.22 (0.11-0.45)***

***P < .001; **P < .01; *P < .05.

In multivariate analysis younger age, higher educational level, greater wealth and greater social capital (being married or cohabiting, high trust and solidarity, lack of safety, higher civic engagement and greater psychological resources) were associated with better cognitive functioning. Further, in multivariate analysis older age and lower social capital (lower social actions, medium safety, lower civic engagement and poorer psychological resources) were associated with physical inactivity (see Table 3).

**Table 3:** Odds ratios for self-reported cognitive functioning and physical inactivity among older South Africans

**Variables**	**Cognitive functioning**		**Physical inactivity**	
***Sociodemographics***	**Unadjusted OR (95% CI)**	**Adjusted OR (95% CI)**	**Unadjusted OR (95% CI)**	**Adjusted OR (95% CI)**
*Age*				
50–59	1.00	1.00	1.00	1.00
60–69	0.58 (0.41-0.81)**	0.59 (0.40-0.89)*	1.67 (1.17-2.38)**	1.71 (1.26-2.32)***
70–79	0.43 (0.31-0.59)***	0.40 (0.26-0.65)***	2.08 (1.27-3.38)**	2.15 (1.33-3.47)**
80+	0.19 (0.10-0.38)***	0.20 (0.08-0.49)***	2.47 (1.59-3.85)***	2.56 (1.37-4.79)**
*Gender*				
Female	1.00	1.00	1.00	1.00
Male	1.52 (1.29-1.80)***	1.25 (0.94-1.67)	0.78 (0.63-0.97)*	0.79 (0.62-1.02)
*Educational level*				
No schooling	1.00	1.00	1.00	---
Less than primary	1.00 (0.69-1.43)	0.91 (0.55-1.49)	0.99 (0.67-1.47)	
Primary	1.32 (0.87-1.99)	1.15 (0.69-1.90)	1.22 (0.82-1.82)	
Secondary	4.05 (2.29-7.15)***	2.96 (1.52-5.77)**	1.00 (0.56-1.78)	
*Wealth*				
Low	1.00	1.00	1.00	---
Medium	1.17 (0.66-2.07)	1.19 (0.69-2.03)	0.93 (0.56-1.55)	
High	2.64 (1.41-4.94)**	2.05 (1.08-3.91)*	1.11 (0.52-2.38)	
***Social capital***				
Married/cohabitating	2.11 (1.58-2.82)***	1.75 (1.27-2.42)***	0.82 (0.64-1.00)	---
Social action				
Low	1.00	1.00	1.00	1.00
Medium	1.38 (0.98-1.94)	1.20 (0.89-1.61)	0.59 (0.39-0.89)*	0.64 (0.44-0.92)*
High	1.45 (1.01-2.09)*	1.42 (0.92-2.19)	0.52 (0.36-0.78)**	0.60 (0.44-0.84)**
Sociability				
Low	1.00	---	1.00	---
Medium	1.33 (0.93-1.93)		0.74 (0.48-1.14)	
High	1.40 (0.76-2.56)		0.75 (0.34-1.69)	
Trust and solidarity				
Low	1.00	1.00	1.00	---
Medium	1.72 (1.27-2.33)***	1.28 (0.88-1.85)	0.82 (0.42-1.63)	
High	2.59 (1.62-4.16)***	2.13 (1.24-3.66)**	0.54 (0.26-1.14)	
Safety				
Low	1.00	1.00	1.00	1.00
Medium	0.52 (0.34-0.79)**	0.53 (0.30-0.95)*	1.85 (1.13-3.02)*	1.92 (1.03-3.59)*
High	0.69 (0.38-1.24)	0.51 (0.23-1.13)	1.07 (0.60-1.92)	1.18 (0.58-2.38)
Civic engagement				
Low	1.00	1.00	1.00	1.00
Medium	1.89 (1.10-3.26)*	2.40 (1.15-5.05)*	0.43 (0.25-0.74)**	0.50 (0.29-0.85)*
High	2.33 (1.28-4.24)**	2.71 (1.27-5.78)*	0.38 (0.22-0.65)***	0.50 (0.27-0.920*
Psychological resources				
Low	1.00	1.00	1.00	1.00
Medium	1.98 (1.42-2.76)***	1.63 (1.17-2.29)**	0.62 (0.42-0.92)*	0.64 (0.33-0.93)*
High	2.86 (1.77-4.62)***	1.97 (1.16-3.33)*	0.38 (0.27-0.54)***	0.46 (0.29-0.72)***

***P < .001; **P < .01; *P < .05.

## Discussion

This paper provides an assessment of the evidence of the association between social capital and health variables. The study reveals significant associations between social capital and a range of health variables, namely: self-rated health, depression, cognitive functioning and physical inactivity. The findings from this national study of older people show that in terms of depressive symptoms, South Africa was similar to America [7,22] and European countries [7,20,21]. These findings include married and cohabiting couples, those with high trust and solidarity, as well as those with medium to high psychological resources all experiencing fewer depressive symptoms. Interestingly all three factors deal with the elderly person having some sort of support from someone else. In terms of females in bivariate analysis exhibiting more depressive symptoms than males, this could be explained from the fact that many females still continue to perform domestic chores at home or vegetable garden while the males retire from all work as well as females having less material support for food and health services [20]. These findings suggest that the characteristics of high social capital could be an essential resource for preventing depressive disorders among South Africans as shown by Putmans social capital conceptualisation. Social clubs may need to be encouraged within communities to engage older people to get out of the home and possibly their routine and socialise. The network flows [7] created within this bridging and bonding socialisation will be an enabler to improved health. Social capital provides people with emotional support and resources for dealing with stressful life events [40]. Programmes for older people that provide emotional, practical and further financial support are indicated [40]. Health promotion programmes might work to improve social capital among older South Africans.

Having access to secondary school and higher also increased one’s self-rated health rating [17,19]. This could be explained by people that have better education, should have access to more money and therefore better health care. Interestingly, it could also be explained by people spending a long time at school and then in a tertiary education system therefore having a long time to build social bonds (bonding social capital) and therefore strengthen their social networks. These strengthened social networks could possibly last a life time and therefore endows people with better health [6,7]. Again, we see that married and cohabiting couples, those with high trust and solidarity, as well as those with medium to high psychological resources all experience better self-rated health.

In terms of cognitive functioning, we see that as age increases, cognitive functioning decreases. This was natural and expected for the majority of populations around the world. Here the results are clearly evident showing that those with better cognitive functioning have higher education, greater wealth, higher trust and solidarity, lower safety, larger civic engagement and more psychological resources. Similar results were also seen in Europe [15,17,20,21,23], and America [22,24]. It was seen in these European and American studies as well as from this South African result that the lack of social capital increased the risk of cognitive decline. It has been widely accepted that physical activity promotes better health [16,19,20,28]. In South Africa, the results of this study show us that the older one gets, physical activity decreases. Further, findings indicate that low social action, lack of safety, low civic engagement and low psychological resources lead to increased physical inactivity. Engaging in society means that one gets to go out and interact with people. Doing things together also eases the task, that is physical activity, at hand and assists in reducing depressive tendencies [15]. National programmess targeting physical inactivity among adults might consider ecological-level interventions that leverage associational involvement and interpersonal relationships to improve population-level physical activity.

### Limitations of the study

This study assesses associations between social capital and health variables based on cross-sectional data. We cannot, therefore, ascribe causality to any of the associated factors in the study. Future studies are needed to examine associations longitudinally using validated social capital measures. The self-report of health variables should be interpreted with caution; it’s possible that measurement errors occurred. Some of the social capital sub-scales (civic engagement, safety, trust and solidarity) had a reliability of below 0.70. Findings relating to these subscales may therefore interpreted with more caution. Possible reasons for the low reliability of these sub-scales could be that different response options were used (Likert type and Yes or No). Finally, data were collected from older adults who were available in the household on the day of the survey. Respondents who were institutionalized (prison, hospital, care home) and not returning to the household within seven days and those who had moved more than 50 kilometers away from the study household were not included.

## Conclusion

Different components of social capital were found to be significantly associated with self-rated poor health, lower depression symptoms, better cognitive functioning and higher physical activity. Interestingly, all the findings above can be conceptualized within the works of Putman. As South Africa develops, we are increasingly finding ourselves ‘Bowling Alone’; our work schedules, high fences and 21^st^ century asocial thinking deprive us of social capital and thus our health. Taking into consideration the limitations of this study mentioned above, this South African study mimics many of the results found in Europe and America. As many of the findings mimics findings from other countries, their tried and tested interventions to increase social capital can be transplanted to South Africa with the relevant testing and modifications for local populations. Social capital among the elder generation in South Africa is imperative for better health.

## Competing interests

The authors declare that they have no competing interests.

## Authors’ contributions

KP designed and analysed the study paper. SR wrote the first draft of the paper. All authors read, contributed and approved the manuscript for publication.

## Pre-publication history

The pre-publication history for this paper can be accessed here:

http://www.biomedcentral.com/1471-2318/13/100/prepub

## Supplementary Material

Additional file 1: Social capital questions. Click here for file
