# Supplementary material for: Social capital and health among older adults in South Africa
Source: BMC Geriatr. 2013 Sep 28;13:100. doi: 10.1186/1471-2318-13-100 (PMC3851859; doi:10.1186/1471-2318-13-100)
Supplement: Additional file 1: — Social capital questions. [file 1471-2318-13-100-S1.docx]

Additional file 1: Social capital questions

We would like to shift away from questions about your direct health. This section of the survey asks your opinions about other areas and issues in your life. The following questions are to get your opinions about community, social and political aspects in your life.

I’d like to start by asking you about the groups or organizations, networks, associations to which you or any member of your household belong. These could be formally organized groups or just groups of people who get together regularly to do an activity or talk about things. As I read the following list of groups, please tell me if anyone in this household belongs to such a group. If yes, tell me which household member is most active in this group, and whether he/she participates actively in the group’s decision making.

We’d like to know about some of your involvement in your community. For all of these, I want you just to give me your best guess.

|  | How often in the last 12 months have you … | Never | Once or twice per year | Once or twice per month | Once or twice per week | Daily |
| --- | --- | --- | --- | --- | --- | --- |
|  | Social action |  |  |  |  |  |
| Q6001 | … attended any public meeting in which there was discussion of local or school affairs? | 1 | 2 | 3 | 4 | 5 |
| Q6002 | … met personally with someone you consider to be a community leader? | 1 | 2 | 3 | 4 | 5 |
| Q6003 | …attended any group, club, society, union or organizational meeting? | 1 | 2 | 3 | 4 | 5 |
| Q6004 | … worked with other people in your neighborhood to fix or improve something? | 1 | 2 | 3 | 4 | 5 |
|  | Sociability |  |  |  |  |  |
| Q6005 | … had friends over to your home? | 1 | 2 | 3 | 4 | 5 |
| Q6006 | … been in the home of someone who lives in a different neighbourhood than you do or had them in your home? | 1 | 2 | 3 | 4 | 5 |
| Q6007 | … socialized with co-workers outside of work? | 1 | 2 | 3 | 4 | 5 |
| Q6009 | … gotten out of the house/your dwelling to attend social meetings, activities, programs or events or to visit friends or relatives? | 1 | 2 | 3 | 4 | 5 |

Trust and solidarityWe’d like to ask you a few questions about how you view other people and institutions.

| Q6012 | Generally speaking, would you say that most people can be trusted or that you can’t be too careful in dealing with people? | 1 Can be trusted  2 Can’t be too careful |
| --- | --- | --- |
| Q6013 | Do you have someone you can trust and confide in? | 1 Yes  2 No |

Next, we’d like to know how much you trust different groups of people.

|  | | To a very great extent | To a great extent | Neither great nor small extent | To a small extent | To a very small extent |
| --- | --- | --- | --- | --- | --- | --- |
| Q6014 | First, think about people in your neighbourhood. Generally speaking, would you say that you can trust them…? | 1 | 2 | 3 | 4 | 5 |
| Q6015 | Now, think about people whom you work with. Generally speaking, would you say that you can trust them …? | 1 | 2 | 3 | 4 | 5 |
| Q6016 | And how about strangers? Generally speaking, would you say that you can trust them …? | 1 | 2 | 3 | 4 | 5 |

SafetyNow we have a few questions about safety in the area where you live.

| Q6017 | In general, how safe from crime and violence do you feel when you are alone at home? | 1 Completely safe  2 Very safe  3 Moderately safe  4 Slightly safe  5 Not safe at all |
| --- | --- | --- |
| Q6018 | How safe do you feel when walking down your street alone after dark? | 1 Completely safe  2 Very safe  3 Moderately safe  4 Slightly safe  5 Not safe at all |
| Q6019 | In the last 12 months, have you or anyone in your household been the victim of a violent crime, such as assault or mugging? | 1 Yes  2 No |

Civic engagementLast, we would like to know about your level of interest in local or national politics and your opinions about how the government responds to issues that interest you. Remember, all responses are confidential.

| Q6020 | How interested would you say you are in politics and national affairs? Would you say you are …..? Read responses | 1 Very interested  2 Interested  3 Neither interested nor uninterested  4 Uninterested  5 Very uninterested |
| --- | --- | --- |
| Q6021 | Lots of people find it difficult to get out and vote. Did you vote in the last state/national/presidential election? | 1 Yes  2 No  97 Refusal |
| Q6022 | How much say do you have in getting the government to address issues that interest you? | 1 Unlimited say  2 A lot of say  3 Some say  4 Little say  5 No say at all |
